# Prevalence and Predictors of Intimate Partner Violence During Pregnancy in Northern Ghana: A Cross‐Sectional Study

**DOI:** 10.1002/hsr2.72351

**Published:** 2026-04-15

**Authors:** Fairuza Marian Salifu, Joseph Lasong, Yula Salifu

**Affiliations:** ^1^ Department of Population and Reproductive Health, School of Public Health University for Development Studies Tamale Ghana; ^2^ Department of Population, Family and Reproductive Health, School of Public Health University of Ghana Legon Ghana

**Keywords:** antenatal care, cross‐sectional study, Ghana, intimate partner violence, predictors, pregnancy

## Abstract

**Background and Aims:**

The persistence of intimate partner violence (IPV) in developing countries is attributable to patriarchal socio‐cultural norms that endorse male dominance, compounded by limited enforcement of legal protections. IPV during pregnancy poses significant risks to both maternal and fetal health. This study aimed to determine the prevalence and predictors of IPV during pregnancy in the Tamale Metropolis, Northern Ghana.

**Methods:**

A hospital‐based cross‐sectional study was conducted from April to May 2023. Systematic random sampling was used to recruit 260 pregnant women from three public health facilities in the Tamale Metropolis. Women with high‐risk pregnancies and non‐consenting participants were excluded. Data were collected using structured questionnaires and analyzed using IBM SPSS version 25. Binary logistic regression was performed to identify independent predictors of IPV. All statistical tests were two‐tailed, with statistical significance set at *p* < 0.05.

**Results:**

The overall prevalence of IPV during the current pregnancy was 60.0%. Compared with women aged ≤ 20 years, those aged 21–30 years (AOR: 0.3; 95% CI: 0.1–0.8; *p* = 0.02) and those aged ≥ 31 years (AOR: 0.2; 95% CI: 0.1–0.6; *p* = 0.01) had significantly lower odds of IPV. Women whose spouses had witnessed their fathers beating their mothers during childhood also had lower adjusted odds of IPV (AOR: 0.3; 95% CI: 0.1–0.9; *p* = 0.03); however, the bivariate analysis showed the opposite direction, and this adjusted finding warrants cautious interpretation and longitudinal investigation.

**Conclusion:**

IPV during pregnancy was highly prevalent in the Tamale Metropolis. Older maternal age was associated with reduced odds of IPV. The inverse adjusted association between spousal childhood exposure to paternal IPV and current IPV risk was unexpected and requires further investigation. Targeted interventions for young pregnant women, integration of IPV screening into antenatal care, and community‐based education campaigns are recommended.

**Abbreviations**
ANC: antenatal care
AOR: adjusted odds ratio
CI: confidence interval
DHS: Demographic and Health Survey
IPV: intimate partner violence
SDG: Sustainable Development Goal
SSA: Sub‐Saharan Africa
VIF: variance inflation factor
WHO: World Health Organization

## 1 Introduction

Intimate partner violence (IPV), encompassing physical, sexual, and emotional abuse by a current or former partner, remains one of the most pervasive public health challenges worldwide [1]. The World Health Organization (WHO) estimates that approximately one in three women experiences IPV during their lifetime [2]. A systematic review and meta‐analysis reported that the pooled prevalence of IPV during pregnancy ranged from approximately 25% to 40% in Sub‐Saharan Africa (SSA) [3]. In Ghana, despite legal frameworks such as the Domestic Violence Act [4], IPV remains a significant concern. National estimates from the Ghana Demographic and Health Survey (DHS) indicate a gradual decline in IPV rates from 34% in 2008 [5] to 30% in 2022 [6]; however, these rates remain disproportionately elevated relative to global targets. Among married or cohabiting women, the prevalence of sexual violence was 15%, while physical violence ranged from 8% in the Greater Accra region to 21% in the Upper East region [6]. IPV rates as high as 47% were recorded in the Savannah region [6]. In contrast, a study conducted in Accra reported that 11.2% of pregnant women experienced some form of IPV [7].

The discrepancy between reported and estimated IPV cases is partly attributable to underreporting, driven by cultural stigma, fear of reprisal, and inadequate awareness of legal rights [2, 8, 9]. Risk factors consistently associated with IPV during pregnancy include younger age, lower educational attainment, and economic dependency [8, 10]. Psychosocial factors—including a partner's substance use, prior exposure to parental violence, and unilateral household decision‐making—have also been associated with elevated IPV risk [9, 11].

IPV during pregnancy is associated with adverse feto‐maternal outcomes, including preterm birth, low birth weight, and pregnancy loss [2, 10]. Persistent IPV is linked to depression, anxiety, and impaired health‐seeking behavior [2]. These consequences impede progress toward Sustainable Development Goal 3 (reduction of maternal and neonatal mortality) and Sustainable Development Goal 5 (gender equality and elimination of violence against women) [12].

In Ghana, the Domestic Violence Act [4] provides legal protection; however, its effectiveness is constrained by enforcement challenges and limited public awareness of available legal recourse. Research indicates that only a minority of IPV cases are officially reported, creating a substantial gap between the actual burden and surveillance data [4, 6]. Most existing studies focus on IPV among women generally, with comparatively few targeting pregnant women specifically. Evidence on IPV during pregnancy in Northern Ghana—a setting characterized by documented socio‐economic disparities, cultural norms endorsing male authority, and limited research on this topic [13]—is particularly scarce.

This study therefore sought to determine the prevalence and predictors of IPV among pregnant women attending selected antenatal care (ANC) clinics in the Tamale Metropolis of Northern Ghana. The findings are intended to inform the development of culturally sensitive, targeted interventions to reduce IPV, improve maternal and fetal health outcomes, and support Ghana's progress toward global health and gender equality targets.

## 2 Methods

### 2.1 Study Area

This study was conducted in the Tamale Metropolis, one of the 16 administrative districts in Ghana's Northern Region. The Metropolis covers a total land area of 646.90 km² and has a population of 233,252, of whom 50.3% are female, and 81% reside in urban areas [14]. Three public health facilities within the Tamale Metropolis were selected: Tamale Teaching Hospital, Tamale Central Hospital, and Tamale West Hospital. The tertiary‐level facility (Tamale Teaching Hospital) was purposively selected on account of its referral role, staff capacity, and large client base, while two secondary‐level hospitals were randomly selected from a pool of four secondary facilities. This random selection of secondary facilities strengthens the representativeness of the study sample by increasing the likelihood of capturing the diversity of patient volumes, staffing levels, and service availability across secondary facilities in the Metropolis.

### 2.2 Study Design

A cross‐sectional study was conducted between April and May 2023 to determine the prevalence and factors associated with IPV among pregnant women in a current partnership. This design was appropriate as it enabled assessment of IPV prevalence and associated factors at a single point in time, consistent with the study's cross‐sectional objectives.

### 2.3 Study Population

Pregnant women attending ANC with ANC record books, in a current partnership of at least 12 months' duration, aged ≥ 18 years, residing in the Tamale Metropolis for at least 6 months, and providing written informed consent were eligible for inclusion. Women with high‐risk pregnancies (including those with placenta previa, severe pre‐eclampsia, or multiple gestations) were excluded because such conditions are associated with specialized clinical management protocols, more frequent hospital visits, and elevated psychological distress—all of which may confound IPV disclosure patterns and introduce information bias. Non‐consenting participants and those absent during the data collection period were also excluded.

### 2.4 Sample Size and Sampling Technique

Sample size was estimated using the Cochran formula: *n* = *Z* ^2^ *α p*(1 − *p*)/*e* ^2^, where *n* = sample size, *Zα* = 1.96 (corresponding to a 95% confidence level), *p* = 0.112 (IPV prevalence among pregnant women in Ghana) [7], and e = 0.05 (acceptable marginal error). Accounting for a 10% non‐response rate, the minimum required sample was 169 participants. A total of 260 participants were enrolled, as recruitment continued for the full planned data collection period across all three facilities, leveraging the complete pool of eligible participants. This exceeded the minimum requirement, thereby improving statistical power, enhancing the precision of prevalence estimates, and strengthening the robustness of subgroup comparisons. Probability proportionate‐to‐size allocation was applied to distribute the sample across the three health facilities (see Supporting Information S1). A sampling frame was constructed from the daily ANC attendance registers at each facility. Systematic sampling with an interval of three was applied, with the starting point randomly selected from the first three entries on each day's register, until the required sample allocation per facility was reached (kindly see Supporting Information S3: Figure 1).

### 2.5 Data Collection and Procedure

Five enumerators with Master of Public Health degrees, fluent in English and relevant local dialects (primarily Dagbani), were recruited and trained for 2 days prior to data collection. The Ghana 2022 DHS questionnaire was adapted for data collection among study participants [6]. Reviewed literature [15, 16] and expert consultation were used to adapt the questionnaire to the local socio‐cultural context. The questionnaire captured: (1) socio‐demographic characteristics of participants and their partners (age, educational level, occupation, religion, ethnicity); (2) IPV‐related items (emotional, physical, and sexual violence); (3) women's empowerment indicators (household decision‐making, financial autonomy); and (4) partner‐level behavioral factors (substance use, sexual behavior) (see Supporting Information S2).

Data were entered into a Google Form to facilitate data capture. Participants who self‐identified as literate in English completed the form independently via a shared link, while enumerators administered the questionnaire orally to non‐literate participants. To ensure data accuracy, enumerators read back each participant's responses for verification, and corrections were made where necessary. Interviews were conducted in English or Dagbani, depending on participant preference.

The experience of IPV during the current pregnancy was the primary outcome variable. “Current IPV” was operationally defined as any experience of emotional, physical, or sexual abuse by an intimate partner occurring during the index pregnancy. Three categories of IPV were assessed—physical (seven items), sexual (eight items), and emotional (nine items)—all pertaining to the current pregnancy (see Supporting Information S2 and Table 4). Women who responded “yes” to at least 1 of the 24 IPV items were coded as having experienced IPV (coded 1), while those who responded “no” to all items were coded as not having experienced IPV (coded 0).

Independent variables included women's and spousal socio‐demographic characteristics, women's empowerment indicators, and spousal behavioral factors, selected based on prior evidence [11, 15, 16]. The questionnaire was piloted among 30 eligible pregnant women at the Tamale Reproductive and Child Health unit and revised for clarity before use in the main study. Interviews lasted approximately 20–45 min. Enumerators were trained to offer breaks as needed to mitigate respondent fatigue, and participants were informed of the estimated duration during the informed consent process.

Ethical approval was obtained from the University for Development Studies Ethics Review Board (UDS/RB/date: 27/02/2023). Administrative permission was obtained from the Northern Regional Health Directorate, the Tamale Metropolitan Health Directorate, and all participating hospital administrations prior to data collection. Written informed consent was obtained from all participants before enrollment. Participation was entirely voluntary; participants were free to decline to answer any question or withdraw at any point without consequence. Confidentiality and anonymity of all data were strictly maintained throughout. The study was conducted in accordance with the principles of the Declaration of Helsinki (2013 revision). The STROBE guidelines for cross‐sectional studies were followed in reporting this study.

### 2.6 Data Analysis

Data exported from Google Forms were cleaned in Microsoft Excel and analyzed using IBM SPSS version 25 (IBM Corp., Armonk, NY, USA). Descriptive statistics were summarized as frequencies and percentages. Chi‐square (*χ*²) tests of independence were performed to assess bivariate associations between IPV and each independent variable; results are reported with the test statistic, degrees of freedom, and *p*‐value. Variables significant at *p* < 0.1 in bivariate analysis were entered as candidates into the multivariable model—this threshold is an exploratory criterion for variable selection and is distinguished from the pre‐specified significance level of *p* < 0.05 for final inference. Binary logistic regression was performed to identify independent predictors of IPV during the current pregnancy, and adjusted odds ratios (AORs) with 95% confidence intervals (CIs) were reported. Multicollinearity was assessed using the variance inflation factor (VIF); all VIF values were below 5, indicating no problematic multicollinearity. Missing data were handled using listwise deletion. A goodness‐of‐fit assessment was performed using the Hosmer–Lemeshow test, which was non‐significant (*p* > 0.05), confirming adequate model fit. All statistical tests were two‐tailed, with a pre‐specified significance level of *p* < 0.05.

## 3 Results

### 3.1 Socio‐Demographic Characteristics of Women

Table 1 presents the socio‐demographic characteristics of participants and their bivariate associations with IPV during the current pregnancy. The mean age was 26 ± 5.3 years. Among participants, 42.3% had no formal education, 22.3% were unemployed, and 86.9% practised Islam.

**Table 1:** Socio‐demographic characteristics of participants and bivariate association with IPV during the current pregnancy (*N* = 260).

Variable	IPV: Yes, *N* (%)	IPV: No, *N* (%)	Total	*χ*²(df); *p*‐value
Age of participant (years)				*χ*²(2) = 8.80; *p* = 0.01*
Mean age: 26 ± 5.3 years				
≤ 20 years	13 (39.4)	20 (60.6)	33	
21–30 years	106 (59.9)	71 (40.1)	177	
≥ 31 years	36 (72.0)	14 (28.0)	50	
Highest educational level				*χ*²(4) = 4.30; *p* = 0.37
No formal education	73 (66.4)	37 (33.6)	110	
Primary	12 (63.2)	7 (36.8)	19	
Junior high school (JHS)	13 (52.0)	12 (48.0)	25	
Senior high school (SHS)	18 (54.5)	15 (45.5)	33	
Tertiary	39 (53.4)	34 (46.6)	73	
Ethnicity				*χ*²(2) = 3.75; *p* = 0.15
Dagomba	123 (58.9)	86 (41.1)	209	
Akan	7 (43.8)	9 (56.3)	16	
Other	25 (71.4)	10 (28.6)	35	
Occupational status				*χ*²(3) = 7.12; *p* = 0.07
Farmer	21 (56.8)	16 (43.2)	37	
Trader	72 (69.2)	32 (30.8)	104	
Unemployed	29 (50.0)	29 (50.0)	58	
Public servant	33 (54.1)	28 (45.9)	61	
Area of residence				*χ*²(1) < 0.001; *p* = 1.00
Rural	25 (59.5)	17 (40.5)	42	
Urban	130 (59.6)	88 (40.4)	218	
Religion				*χ*²(1) = 3.90; *p* = 0.06
Christianity	15 (44.1)	19 (55.9)	34	
Islam	140 (61.9)	86 (38.1)	226	

*Note:* Percentages are row proportions within each variable category. IPV = intimate partner violence during the current pregnancy.

* *p* < 0.05.

In the bivariate analysis, women's age was the only socio‐demographic variable significantly associated with IPV (*χ*²(2) = 8.80; *p* = 0.01). Among women aged ≥ 31 years, 72.0% experienced IPV during the current pregnancy; the corresponding figures were 59.9% among women aged 21–30 years and 39.4% among those aged ≤ 20 years. No significant bivariate associations were observed for educational status (*χ*²(4) = 4.30; *p* = 0.37), ethnicity (*χ*²(2) = 3.75; *p* = 0.15), occupational status (*χ*²(3) = 7.12; *p* = 0.07), area of residence (*χ*²(1) < 0.001; *p* = 1.00), or religion (*χ*²(1) = 3.90; *p* = 0.06) (Table 1).

### 3.2 Spousal Characteristics

Table 2 presents spousal characteristics and their bivariate associations with IPV. In the bivariate analysis, spousal ethnicity (*χ*²(2) = 7.87; *p* = 0.02), having children with another woman (*χ*²(1) = 6.03; *p* = 0.02), spousal smoking (*χ*²(1) = 7.46; *p* = 0.01), a prior marriage or divorce (*χ*²(1) = 6.61; *p* = 0.01), multiple sexual partners (*χ*²(1) = 6.32; *p* = 0.02), and spousal childhood exposure to paternal IPV (*χ*²(1) = 8.26; *p* < 0.01) were each significantly associated with IPV during the current pregnancy. Among women whose spouses reported witnessing their father beating their mother during childhood, 81.1% experienced IPV during the current pregnancy, compared with 56.1% of women whose spouses reported no such exposure—a bivariate direction consistent with social learning theory [17, 18]. The direction of this association reversed in the adjusted analysis, discussed further in Section Conclusion, 4 (Table 2).

**Table 2:** Spousal characteristics and bivariate association with IPV during the current pregnancy (*N* = 260).

Variable	IPV: Yes, *N* (%)	IPV: No, *N* (%)	Total	*χ*²(df); *p* value
Age of spouse (years)				*χ*²(2) = 2.57; *p* = 0.28
≤ 25	7 (41.2)	10 (58.8)	17	
26–46	140 (60.9)	90 (39.1)	230	
> 46	8 (61.5)	5 (38.5)	13	
Highest educational level				*χ*²(2) = 4.49; *p* = 0.11
No formal education	68 (66.0)	35 (34.0)	103	
Middle school	9 (42.9)	12 (57.1)	21	
Tertiary	78 (57.4)	58 (42.6)	136	
Ethnicity				*χ*²(2) = 7.87; *p* = 0.02*
Dagomba	141 (62.7)	84 (37.3)	225	
Gonja	6 (54.5)	5 (45.5)	11	
Other	8 (33.3)	16 (66.7)	24	
Occupation				*χ*²(3) = 2.00; *p* = 0.41
Farmer	63 (65.6)	33 (34.4)	96	
Trader	12 (54.5)	10 (45.5)	22	
Unemployed	5 (45.5)	6 (54.5)	11	
Public servant	75 (57.3)	56 (42.7)	131	
Religion				*χ*²(1) = 0.94; *p* = 0.34
Christianity	16 (51.6)	15 (48.4)	31	
Islam	139 (60.7)	90 (39.3)	229	
Spouse has children with another woman				*χ*²(1) = 6.03; *p* = 0.02*
Yes	50 (72.5)	19 (27.5)	69	
No	105 (55.6)	84 (44.4)	189	
Spouse smokes				*χ*²(1) = 7.46; *p* = 0.01*
Yes	35 (77.8)	10 (22.2)	45	
No	120 (55.8)	95 (44.2)	215	
Spouse drinks alcohol				*χ*²(1) = 1.19; *p* = 0.32
Yes	20 (69.0)	9 (31.0)	29	
No	135 (58.4)	96 (41.6)	231	
Spouse has a prior marriage or divorce				*χ*²(1) = 6.61; *p* = 0.01*
Yes	23 (82.1)	5 (17.9)	28	
No	132 (56.9)	100 (43.1)	232	
Spouse has multiple sexual partners				*χ*²(1) = 6.32; *p* = 0.02*
Yes	60 (70.6)	25 (29.4)	84	
No	95 (54.3)	80 (45.7)	175	
How a woman responds to disagreements with her spouse				*χ*²(2) = 5.10; *p* = 0.08
Keeps calm	74 (55.2)	60 (44.8)	134	
Talks back	40 (72.7)	15 (27.3)	55	
Apologizes	41 (57.7)	30 (42.3)	71	
Spouse witnessed his father beating his mother during childhood				*χ*²(1) = 8.26; *p* < 0.01*
Yes	30 (81.1)	7 (18.9)	37	
No	125 (56.1)	98 (43.9)	223	

*Note:* Percentages are row proportions within each variable category.

* *p* < 0.05.

### 3.3 Women's Empowerment Characteristics

Table 3 presents women's empowerment indicators and their bivariate associations with IPV. Decision‐making on large household purchases by the spouse alone (*χ*²(2) = 7.83; *p* = 0.02), decision‐making on visiting family by the participant alone (*χ*²(2) = 8.25; *p* = 0.02), and spousal control of contraceptive decisions (*χ*²(2) = 13.29; *p* = 0.001) were significantly associated with IPV in the bivariate analysis. Decision‐making on daily household purchases also reached significance (*χ*²(2) = 6.40; *p* = 0.04) (Table 3).

**Table 3:** Women's empowerment characteristics and bivariate association with IPV during the current pregnancy (*N* = 260).

Variable	IPV: Yes, *N* (%)	IPV: No, *N* (%)	Total	*χ*²(df); *p* value
Who decides on the participant's healthcare?				*χ*²(2) = 5.38; *p* = 0.07
Participant alone	41 (66.1)	21 (33.9)	62	
Spouse alone	97 (61.0)	62 (39.0)	159	
Both jointly	17 (43.6)	22 (56.4)	39	
Who decides on large household purchases?				*χ*²(2) = 7.83; *p* = 0.02*
Participant alone	35 (71.4)	14 (28.6)	49	
Spouse alone	101 (60.5)	66 (39.5)	167	
Both jointly	19 (43.2)	25 (56.8)	44	
Who decides on daily household purchases?				*χ*²(2) = 6.40; *p* = 0.04*
Participant alone	40 (71.4)	16 (28.6)	56	
Spouse alone	94 (59.1)	65 (40.9)	159	
Both jointly	21 (46.7)	24 (53.3)	45	
Who decides on visiting family or relatives?				*χ*²(2) = 8.25; *p* = 0.02*
Participant alone	62 (68.1)	29 (31.9)	91	
Spouse alone	77 (59.2)	53 (40.8)	130	
Both jointly	16 (41.0)	23 (59.0)	39	
Does the participant have control over her own earnings?				*χ*²(1) = 0.05; *p* = 0.87
Yes	130 (59.9)	87 (40.1)	217	
No	25 (58.1)	18 (41.9)	43	
Does the participant have control over the spouse's earnings?				*χ*²(1) = 1.11; *p* = 0.42
Yes	7 (46.7)	8 (53.3)	15	
No	148 (60.4)	97 (39.6)	245	
Does the participant know the amount the spouse earns?				*χ*²(1) = 3.00; *p* = 0.09
Yes	54 (52.9)	48 (47.1)	102	
No	100 (63.7)	57 (36.3)	157	
Who decides on contraceptive use?				*χ*²(2) = 13.29; *p* = 0.001*
Participant alone	57 (69.5)	25 (30.5)	82	
Spouse alone	21 (38.9)	33 (61.1)	54	
Both jointly	77 (62.1)	47 (37.9)	124	
Who decides on the number of children?				*χ*²(2) = 5.43; *p* = 0.07
Participant alone	6 (54.5)	5 (45.5)	11	
Spouse alone	48 (71.6)	19 (28.4)	67	
Both jointly	101 (55.5)	81 (44.5)	182	

*Note:* Percentages are row proportions within each variable category.

* *p* < 0.05.

### 3.4 Prevalence of IPV During Pregnancy

Overall, 60.0% (95% CI: 54.1%–65.9%) of women experienced at least one form of IPV during the current pregnancy (*n* = 156/260). Emotional violence was the most prevalent form, followed by sexual violence, with physical violence recorded at the lowest prevalence of the three types (kindly see Supporting Information S4: Figure 2).

### 3.5 Types of IPV During Pregnancy

#### 3.5.1 Physical Violence

Among participants who experienced IPV during the current pregnancy (*n* = 156), the most frequently reported physical act was being slapped (55.1%), followed by being threatened or attacked with a weapon (5.1%) and being punched with a fist or object (4.5%). Other physical acts were each reported by approximately 3%–4% of affected women (Table 4).

**Table 4:** Forms of intimate partner violence reported by participants during the current pregnancy (*n* = 156*).

Item	Yes, *N* (%)	No, *N* (%)
Physical violence—“During this pregnancy, has your partner ever…”		
Slapped you	86 (55.1)	70 (44.9)
Twisted your arm or pulled your hair	6 (3.8)	150 (96.2)
Pushed, shook, or thrown something at you	5 (3.2)	151 (96.8)
Punched you with his fist or with something that could hurt you	7 (4.5)	149 (95.5)
Kicked, dragged, or beaten you up	5 (3.2)	151 (96.8)
Tried to choke or burn you on purpose	5 (3.2)	151 (96.8)
Threatened or attacked you with a knife, gun, or any other weapon	8 (5.1)	148 (94.9)
Emotional violence—“During this pregnancy, has your partner ever…”		
Humiliated or embarrassed you in front of others	141 (90.4)	15 (9.6)
Threatened you with harm	24 (15.4)	132 (84.6)
Insulted you or made you feel bad about yourself	103 (66.0)	53 (34.0)
Kept you from seeing or talking to your family or friends	16 (10.3)	140 (89.7)
Kept you from having access to a job, money, or financial resources	13 (8.3)	143 (91.7)
Mistrusted your emotions or accused you unfairly	54 (34.6)	102 (65.4)
Accused you of flirting or cheating and blamed you for their problems	33 (21.2)	123 (78.8)
Made you feel constantly confused and trapped, such that you could not express your emotions	84 (53.8)	72 (46.2)
Made you feel like you were “walking on eggshells” around him	25 (16.0)	131 (84.0)
Sexual violence—“During this pregnancy, has your partner ever…”		
Physically forced you into unwanted sexual intercourse	61 (39.1)	95 (60.9)
Forced you into other unwanted sexual acts	13 (8.3)	143 (91.7)
Made you agree to have sex only because you were afraid	19 (12.2)	137 (87.8)
Refused to use or agree to safe sex practices	25 (16.0)	131 (84.0)
Forced his decisions regarding birth control, pregnancy, or abortion on you	55 (35.3)	101 (64.7)
Withheld sex as a form of punishment	26 (16.7)	130 (83.3)
Persisted with sexual advances after you said “no”	131 (83.97)	25 (16.0)
Insulted or criticized your body or private parts	133 (85.3)	23 (14.7)

*Note:* Percentages are column proportions within this IPV‐positive subsample.

* Denominator = 156 women who reported experiencing any form of IPV during the current pregnancy.

#### 3.5.2 Emotional Violence

Humiliation was the most prevalent form of emotional abuse, reported by 90.4% of women who experienced IPV during the current pregnancy. Other commonly endorsed items included being insulted or made to feel bad about oneself (66.0%), feeling constantly confused and trapped such that emotions could not be expressed (53.8%), and being mistrusted or unfairly accused (34.6%). Threats of harm were reported by 15.4%, and social isolation (being kept from family or friends) by 10.3% (Table 4).

#### 3.5.3 Sexual Violence

Insulting or criticizing the participant's body was the most reported sexual IPV act (85.3%), followed by persisting with sexual advances after the participant refused (84.0%). Physical force into unwanted intercourse was reported by 39.1% of affected women, while forced reproductive decision‐making occurred in 35.3%. Withholding sex as punishment (16.7%), refusing safe sex (16.0%), and agreeing to sex out of fear (12.2%) were also endorsed (Table 4).

### 3.6 Predictors of IPV Among Pregnant Women

Table 5 presents the results of the multivariable binary logistic regression analysis. After adjusting for all variables simultaneously, two factors were independently and statistically significantly associated with IPV during the current pregnancy: maternal age and spousal childhood exposure to paternal IPV.

**Table 5:** Multivariable predictors of intimate partner violence during the current pregnancy—Binary logistic regression (*N* = 260).

Variable	IPV: Yes, *N* (%)	IPV: No, *N* (%)	Total	AOR (95% CI)	*p* value
Maternal age (years)					
≤ 20 years (Reference)	13 (39.4)	20 (60.6)	33	1.0 (Reference)	—
21–30 years	106 (59.9)	71 (40.1)	177	**0.3 (0.1–0.8)**	**0.02**
≥ 31 years	36 (72.0)	14 (28.0)	50	**0.2 (0.1–0.6)**	**0.01**
Spousal ethnicity					
Dagomba	141 (62.7)	84 (37.3)	225	0.4 (0.1–1.1)	0.07
Gonja	6 (54.5)	5 (45.5)	11	0.4 (0.1–2.1)	0.27
Other (Reference)	8 (33.3)	16 (66.7)	24	1.0 (Reference)	—
Spouse has children with another woman					
Yes	50 (72.5)	19 (27.5)	69	0.9 (0.4–2.1)	0.89
No (Reference)	105 (55.6)	84 (44.4)	189	1.0 (Reference)	—
Spouse smokes					
Yes	35 (77.8)	10 (22.2)	45	0.8 (0.3–2.0)	0.63
No (Reference)	120 (55.8)	95 (44.2)	215	1.0 (Reference)	—
Spouse has a prior marriage or divorce					
Yes	23 (82.1)	5 (17.9)	28	0.5 (0.1–1.5)	0.20
No (Reference)	132 (56.9)	100 (43.1)	232	1.0 (Reference)	—
Spouse has multiple sexual partners					
Yes	60 (70.6)	25 (29.4)	84	0.8 (0.1–0.9)	0.64
No (Reference)	95 (54.3)	80 (45.7)	175	1.0 (Reference)	—
Spouse witnessed his father beating his mother during childhood					
Yes	30 (81.1)	7 (18.9)	37	**0.3 (0.1–0.9)**	**0.03**
No (Reference)	125 (56.1)	98 (43.9)	223	1.0 (Reference)	—
Decision‐maker for large household purchases					
Participant alone	35 (71.4)	14 (28.6)	49	0.4 (0.03–5.1)	0.47
Spouse alone	101 (60.5)	66 (39.5)	167	0.5 (0.01–3.3)	0.45
Both jointly (Reference)	19 (43.2)	25 (56.8)	44	1.0 (Reference)	—
Decision‐maker for daily household purchases					
Participant alone	40 (71.4)	16 (28.6)	56	1.3 (0.0–15.1)	0.81
Spouse alone	94 (59.1)	65 (40.9)	159	1.8 (0.3–12.0)	0.57
Both jointly (Reference)	21 (46.7)	24 (53.3)	45	1.0 (Reference)	—
Decision‐maker for visiting family or relatives					
Participant alone	62 (68.1)	29 (31.9)	91	0.8 (0.3–2.2)	0.63
Spouse alone	77 (59.2)	53 (40.8)	130	1.0 (0.3–3.0)	0.97
Both jointly (Reference)	16 (41.0)	23 (59.0)	39	1.0 (Reference)	—
Decision‐maker for contraceptive use					
Participant alone	57 (69.5)	25 (30.5)	82	0.7 (0.3–1.3)	0.23
Spouse alone	21 (38.9)	33 (61.1)	54	2.0 (0.9–4.2)	0.06
Both jointly (Reference)	77 (62.1)	47 (37.9)	124	1.0 (Reference)	—

*Note:* Bold values indicate statistical significance at *p* < 0.05. Hosmer–Lemeshow test: *p* > 0.05 (adequate model fit). VIF values all < 5 (no multicollinearity). All tests two‐tailed. Reference categories in parentheses.

Abbreviations: AOR, adjusted odds ratio; CI, confidence interval.

Compared with women aged ≤ 20 years, those aged 21–30 years had approximately 70% lower adjusted odds of experiencing IPV (AOR: 0.3; 95% CI: 0.1–0.8; *p* = 0.02), and those aged ≥ 31 years had approximately 80% lower adjusted odds (AOR: 0.2; 95% CI: 0.1–0.6; *p* = 0.01). Women whose spouses reported having witnessed their father beating their mother during childhood had approximately 70% lower adjusted odds of experiencing IPV compared with those whose spouses reported no such exposure (AOR: 0.3; 95% CI: 0.1–0.9; *p* = 0.03). This adjusted inverse association contrasts with the bivariate pattern, in which spousal childhood exposure was associated with substantially higher IPV prevalence (81.1% vs. 56.1%). The remaining variables entered into the model were not independently significant at *p* < 0.05 in the adjusted analysis (Table 5).

## 4 Discussion

This study determined the prevalence and predictors of IPV during pregnancy among women attending ANC facilities in the Tamale Metropolis, Northern Ghana. The overall prevalence was 60.0%, substantially higher than both national (30%) and regional SSA estimates. Two independent predictors were identified in the adjusted analysis: maternal age—inversely associated with IPV—and spousal childhood exposure to paternal IPV, whose adjusted inverse association contrasted with its positive bivariate direction, indicating a complex relationship requiring further investigation.

The prevalence observed in this study exceeds IPV rates reported in prior studies from Ghana (30.5%) [15] and Nigeria (31.5%) [19] and is also higher than pooled estimates from SSA (52%–54%) [20, 21]. Substantially lower rates of 2.2%–8.9% have been reported in high‐income settings [22, 23]. These differences likely reflect variability in socio‐cultural contexts, study populations, measurement tools, operational definitions of IPV, and reporting behaviors. The high prevalence in this setting is consistent with evidence of elevated IPV burden in Northern Ghana, where patriarchal norms and limited awareness of legal protections persist [13]. These findings underscore a critical gap in IPV prevention and response—particularly for pregnant women—and emphasize the need to integrate systematic IPV screening into ANC.

Older maternal age was independently associated with reduced odds of IPV during pregnancy. This is consistent with prior research indicating that younger women may face heightened vulnerability, potentially due to greater economic dependency, more limited relationship negotiating capacity, and less developed social networks [24, 25]. Although some studies present conflicting evidence [26], inconsistencies likely reflect contextual differences in study populations and analytical approaches. It is plausible that older age is associated with greater economic independence and stronger relationship‐negotiating capacity, both of which are empirically associated with lower IPV risk in some contexts. However, these represent potential explanatory pathways warranting empirical testing in longitudinal research and should not be interpreted as established causal mechanisms. These findings highlight young pregnant women as a priority group for targeted intervention.

The multivariable model identified that women whose spouses had witnessed their fathers beating their mothers during childhood had approximately 70% lower adjusted odds of experiencing IPV (AOR: 0.3; 95% CI: 0.1–0.9; *p* = 0.03). This finding is counterintuitive relative to social learning theory, which posits that childhood exposure to domestic violence increases the risk of perpetration and victimization in adulthood [10, 17, 18]. Critically, the bivariate analysis showed the opposite direction: women whose spouses had witnessed paternal IPV had substantially higher IPV prevalence (81.1% vs. 56.1%; *χ*²(1) = 8.26; *p* < 0.01), consistent with social learning theory. The reversal in direction after multivariable adjustment may reflect a suppressor variable effect, whereby the association between spousal childhood exposure and current IPV is modified by co‐variables simultaneously controlled in the model—such as spousal ethnicity or relationship characteristics—that were not independently significant but may collectively alter the direction of this association. Measurement heterogeneity in respondents' interpretation of “witnessing paternal IPV” may also have contributed. Protective factors such as psychosocial resilience or higher educational attainment, not captured in this study, may further complicate the relationship. This finding should be interpreted with considerable caution. It does not establish a protective mechanism. Rigorous longitudinal and qualitative research is warranted to investigate this complex association.

Overall, these findings emphasize the multidimensional nature of IPV risk during pregnancy and the need for context‐sensitive, multi‐level interventions targeting individual vulnerabilities and broader socio‐cultural determinants.

## 5 Strengths and Limitations

This study addresses a documented evidence gap on IPV during pregnancy in Northern Ghana, a setting with limited prior research. Key methodological strengths include: use of a validated, contextually adapted instrument (Ghana DHS questionnaire); multi‐facility sampling across one tertiary and two randomly selected secondary facilities, improving sample representativeness; a clearly defined and operationalised outcome measure; and adherence to STROBE reporting guidelines. The study provides empirical prevalence estimates and identifies two independent predictors—maternal age and spousal childhood IPV exposure—to guide intervention targeting.

Several limitations should be considered. First, reliance on self‐reported data raises concerns about social desirability bias and potential underreporting of IPV; future studies should consider multi‐informant methods to improve disclosure. Second, the cross‐sectional design precludes causal inference regarding identified associations; longitudinal designs are needed to establish temporal relationships. Third, the facility‐based, single‐metropolis sampling restricts generalizability to the broader Ghanaian pregnant population; future research should employ population‐based, multi‐regional sampling. Fourth, the absence of knowledge, attitude, and perception measures limits understanding of the social and cognitive drivers of IPV reporting; incorporating these domains would enrich future investigations. Fifth, the study excluded women with high‐risk pregnancies, which may limit applicability to this subpopulation.

## 6 Conclusions

This study documented a high prevalence of IPV during pregnancy (60.0%) in the Tamale Metropolis, Northern Ghana. Older maternal age was independently associated with substantially lower odds of IPV, underscoring young pregnant women as a priority target for prevention. The unexpected inverse adjusted association between spousal childhood exposure to paternal IPV and current IPV contrasts with the bivariate direction and requires cautious interpretation pending longitudinal research.

Based on these findings, the following responses are recommended. First, routine IPV screening and brief counseling should be integrated into ANC across all public health facilities, with prioritized attention to young women; this approach is supported by WHO guidance on IPV identification in health settings. Second, community‐based education programmes engaging men and boys should challenge norms that normalize IPV and promote gender‐equitable relationship models. Third, psychosocial support employing trauma‐informed approaches—defined here as care that acknowledges and responds to the impact of prior trauma on current health behavior and relationships—should be available for affected women and high‐risk couples. Fourth, district health directorates should strengthen institutional frameworks for routine IPV data collection and monitoring. Finally, longitudinal and mixed‐methods research is recommended to generate robust evidence on the mechanisms and trajectories of IPV during pregnancy in this and comparable settings.

## Author Contributions

F.M.S., Y.S., and J.L. conceptualized the research plan. F.M.S. led data collection. Y.S. and J.L. conducted statistical analyses, interpreted the results, and drafted the manuscript. F.M.S., Y.S., and J.L. reviewed and edited the manuscript. All authors approved the final version.

## Funding

The authors have nothing to report.

## Ethics Statement

Ethical approval was obtained from the University for Development Studies Ethics Review Board (UDS/RB/date: 27/02/2023). Administrative permission was obtained from the Northern Regional Health Directorate, the Tamale Metropolitan Health Directorate, and all participating hospital administrations. Written informed consent was obtained from all participants before enrollment. Participation was voluntary; participants could withdraw or decline to answer any question without consequence. The study was conducted in accordance with the principles of the Declaration of Helsinki (2013 revision).

## Consent

The authors have nothing to report.

## Conflicts of Interest

The authors declare no conflicts of interest.

## Transparency Statement

The lead author, Joseph Lasong, affirms that this manuscript is an honest, accurate, and transparent account of the study being reported; that no important aspects of the study have been omitted; and that any discrepancies from the study as planned (and, if relevant, registered) have been explained.

## Supporting information

Supporting File 1

Supporting File 2

Supporting File 3

Supporting File 4

Supporting File 5

## Data Availability

All data generated or analyzed during this study are available upon reasonable request to the corresponding author (jlasong@uds.edu.gh). No publicly archived dataset was created or used.
