# Supplementary material for: Prevalence and Predictors of Intimate Partner Violence During Pregnancy in Northern Ghana: A Cross‐Sectional Study
Source: Health Sci Rep. 2026 Apr 15;9(4):e72351. doi: 10.1002/hsr2.72351 (PMC13083581; doi:10.1002/hsr2.72351)
Supplement: Supplementary file 1 — Supporting File 1 [file HSR2-9-e72351-s003.docx]

**STROBE STATEMENT — COMPLETED CHECKLIST**

**Cross-Sectional Study Reporting Checklist**

*Study: Prevalence and predictors of intimate partner violence during pregnancy in Northern Ghana: a cross-sectional study*

Authors: Fairuza Marian Salifu, Joseph Lasong, Yula Salifu

| **STROBE CHECKLIST FOR CROSS-SECTIONAL STUDIES** | | | |
| --- | --- | --- | --- |
| **Section/Topic** | **Item** | **Recommendation** | **Page No.** |
| **TITLE AND ABSTRACT** | | | |
| **Title and abstract** | **1** | (a) Indicate the study's design with a commonly used term in the title or the abstract | **1** |
|  |  | (b) Provide in the abstract an informative and balanced summary of what was done and what was found | **1–2** |
| **INTRODUCTION** | | | |
| **Background/rationale** | **2** | Explain the scientific background and rationale for the investigation being reported | **2–3** |
| **Objectives** | **3** | State specific objectives, including any prespecified hypotheses | **3** |
| **METHODS** | | | |
| **Study design** | **4** | Present key elements of study design early in the paper | **4** |
| **Setting** | **5** | Describe the setting, locations, and relevant dates, including periods of recruitment, exposure, follow-up, and data collection | **4** |
| **Participants** | **6a** | (a) Give the eligibility criteria, and the sources and methods of selection of participants | **4–5** |
|  | **6b** | (b) For matched studies: give matching criteria and number of exposed and unexposed — Not applicable (cross-sectional study) | N/A |
| **Variables** | **7** | Clearly define all outcomes, exposures, predictors, potential confounders, and effect modifiers. Give diagnostic criteria, if applicable | **5–6** |
| **Data sources/measurement** | **8*** | For each variable of interest, give sources of data and details of methods of assessment (measurement). Describe comparability of assessment methods if there is more than one group | **5–6** |
| **Bias** | **9** | Describe any efforts to address potential sources of bias | **5–6** |
| **Study size** | **10** | Explain how the study size was arrived at | **5** |
| **Quantitative variables** | **11** | Explain how quantitative variables were handled in the analyses. If applicable, describe which groupings were chosen and why | **6** |
| **Statistical methods** | **12a** | (a) Describe all statistical methods, including those used to control for confounding | **6** |
|  | **12b** | (b) Describe any methods used to examine subgroups and interactions | **6** |
|  | **12c** | (c) Explain how missing data were addressed | **6** |
|  | **12d** | (d) If applicable, describe analytical methods taking account of sampling strategy | **6** |
|  | **12e** | (e) Describe any sensitivity analyses | N/A |
| **Participants** | **13a*** | (a) Report numbers of individuals at each stage of the study (e.g., numbers potentially eligible, examined for eligibility, confirmed eligible, enrolled, completing follow-up, and analysed) | **Flow Diagram** |
|  | **13b** | (b) Give reasons for non-participation at each stage | **Flow Diagram** |
|  | **13c** | (c) Consider use of a flow diagram | **Flow Diagram** |
| **RESULTS** | | | |
| **Descriptive data** | **14a*** | (a) Give characteristics of study participants (e.g., demographic, clinical, social) and information on exposures and potential confounders | **7–9** |
|  | **14b** | (b) Indicate number of participants with missing data for each variable of interest | **6** |
| **Outcome data** | **15*** | Report numbers of outcome events or summary measures | **7–9** |
| **Main results** | **16a** | (a) Give unadjusted estimates and, if applicable, confounder-adjusted estimates and their precision (e.g., 95% CI). Make clear which confounders were adjusted for and why | **9–10** |
|  | **16b** | (b) Report category boundaries when continuous variables were categorised | **7–9** |
|  | **16c** | (c) If relevant, consider translating estimates of relative risk into absolute risk for a meaningful time period | N/A |
| **Other analyses** | **17** | Report other analyses done — e.g., analyses of subgroups and interactions, and sensitivity analyses | N/A |
| **DISCUSSION** | | | |
| **Key results** | **18** | Summarise key results with reference to study objectives | **11–12** |
| **Limitations** | **19** | Discuss limitations of the study, taking into account sources of potential bias or imprecision. Discuss both direction and magnitude of any potential bias | **13** |
| **Interpretation** | **20** | Give a cautious overall interpretation of results considering objectives, limitations, multiplicity of analyses, results from similar studies, and other relevant evidence | **11–14** |
| **Generalisability** | **21** | Discuss the generalisability (external validity) of the study results | **13** |
| **OTHER INFORMATION** | | | |
| **Funding** | **22** | Give the source of funding and the role of the funders for the present study and, if applicable, for the original study on which the present article is based | **15** |
| **Notes:** (*) Give information separately where applicable. "N/A" = not applicable to this cross-sectional study. This checklist is completed per the STROBE Statement for Cross-Sectional Studies. Available at: www.strobe-statement.org. This checklist should be submitted as a supplementary document. | | | |

**Reference:** von Elm E, Altman DG, Egger M, Pocock SJ, Gøtzsche PC, Vandenbroucke JP; STROBE Initiative. The Strengthening the Reporting of Observational Studies in Epidemiology (STROBE) statement: guidelines for reporting observational studies. *Lancet.* 2007;370(9596):1453-7. doi:10.1016/S0140-6736(07)61602-X
