# Supplementary material for: Prevalence and Predictors of Intimate Partner Violence During Pregnancy in Northern Ghana: A Cross‐Sectional Study
Source: Health Sci Rep. 2026 Apr 15;9(4):e72351. doi: 10.1002/hsr2.72351 (PMC13083581; doi:10.1002/hsr2.72351)
Supplement: Supplementary file 2 — Supporting File 2 [file HSR2-9-e72351-s002.docx]

The average monthly antenatal care attendance for the Tamale Teaching Hospital (TTH), Tamale West Hospital (TWH) and Tamale Central Hospital (TCH) according to each facilities record are 200, 120, and 170 respectively.

The total sample size was 260

Thus, total average monthly antenatal care attendance for all facilities were 490.

Therefore, the sample size estimated for each facility was:

TTH= 200/490 X 260 = 106

TWH= 120/490 X 260 = 64

TCH= 170/490 X 260 = 90
