# Supplementary material for: Prevalence and Predictors of Intimate Partner Violence During Pregnancy in Northern Ghana: A Cross‐Sectional Study
Source: Health Sci Rep. 2026 Apr 15;9(4):e72351. doi: 10.1002/hsr2.72351 (PMC13083581; doi:10.1002/hsr2.72351)
Supplement: Supplementary file 3 — Supporting File 3 [file HSR2-9-e72351-s005.docx]

**Questionnaire**

| **BACKGROUND INFORMATON** | |
| --- | --- |
| Name of Interviewer |  |
| Date of Interview |  |
| Respondent ID |  |
| ANC card number |  |

| **Q#** | **Question** | **Responds** | **code** |
| --- | --- | --- | --- |
|  | **Section A: Socio-demographics** | |  |
| 1 | What is your age (in years)? | ……………………. |  |
| 2 | What is the highest level of education of respondents? | 1. No education  2. Primary  3. Junior High School  4. Senior High School  5. Tertiary  6. Informal education |  |
| 3 | What is your marital status? | 1. Single  2. Married  3. cohabitation  4. Divorced/separated  5. Widowed |  |
| 4 | What is your ethnicity? | 1. Dagomba  2. Mamprusi  3. Gonja  4.Akan  4. Others specify ………………… |  |
| 5 | What is your occupation? | 1. Farmer  2. Trader  3. Unemployed  4. Public servant  5. Others specify ……………………. |  |
| 6 | What is your monthly income? | GHS…………………. |  |
| 7 | What is your area of residence? | 1. Rural  2. Urban  3. Peri urban |  |
| 8 | What is your household size? | ………………………………. |  |
| 9 | What is your religion? | 1. Christianity  2. Muslim  3. Traditionalist  4. Others specify |  |
| 10 | How many pregnancies have you had? |  |  |
| 11 | How many children do you have? | ………………………….. |  |

**Section B: Prevalence and type of violence exerted by intimate partners**

12. Have you ever experienced domestic violence from your partner? a. Yes b. No

Which of the following listed statements have you ever experienced from your partner?

| **No.** | **Statement** | Yes | No |
| --- | --- | --- | --- |
|  | **Physical violence** |  |  |
| 13 | Ever been slapped |  |  |
| 14 | Ever been twisted in your arm or pulled your hair |  |  |
| 15 | Ever been pushed, shook, or threw something at you |  |  |
| 16 | Ever been punched with his fist or with something that could hurt you |  |  |
| 17 | Ever been kicked, dragged or beaten up |  |  |
| 18 | Ever been tried to choke you or burn you on purpose |  |  |
| 19 | Ever been threatened or attacked with a knife, gun, or any other weapon |  |  |
| 20 | How often do you experience any of these? a. Always [ ] b. at least once every week [ ] c. at least once every month [ ] d. sometimes [ ] |  |  |
|  | **Sexual violence** |  |  |
| 21 | Ever been physically forced into unwanted sex? |  |  |
| 22 | Ever been forced into other unwanted sexual act? |  |  |
| 23 | **You agree to have sex only because you’re afraid** |  |  |
| 24 | **He refuses to practice safe sex** |  |  |
| 25 | **He forces his decisions of birth control, pregnancy and/or abortion** |  |  |
| 26 | **He withholds sex as punishment** |  |  |
| 27 | **He persists even after you’ve said “no”** |  |  |
| 28 | **He insults and criticizes your body** |  |  |
| 29 | How often do you experience any of these? a. Always [ ] b. at least once every week [ ] c. at least once every month [ ] d. sometimes [ ] |  |  |
|  | **Emotional violence** |  |  |
| 30 | Ever been humiliated |  |  |
| 31 | Ever been threatened with harm |  |  |
| 32 | Ever been insulted or made to feel bad |  |  |
| 33 | Ever been kept from seeing or talking to your family or friends |  |  |
| 34 | Ever been kept from having access to a job, money or financial resources |  |  |
| 35 | Your partner mistrusts you and your emotions |  |  |
| 36 | Accuse you of flirting or cheating and blaming you for their problems |  |  |
| 37 | You feel constantly confused and trapped because you can’t express your emotions and opinions. |  |  |
| 38 | You walk on eggshells |  |  |
| 39 | How often do you experience any of these? a. Always [ ] b. at least once every week [ ] c. at least once every month [ ] d. sometimes [ ] |  |  |

**Section C: Risk factors associated with violence among pregnant women**

| **Q#** | **Question** | **Responds** | **code** |
| --- | --- | --- | --- |
| 40 | What is your spouse’s age (in years)? | ……………………. |  |
| 41 | What is your spouse’s highest level of education of respondents? | 1. No education  2. Primary  3. Junior High School  4. Senior High School  5. Tertiary  6. Informal education |  |
| 42 | What is your spouse’s ethnicity? | 1. Dagomba  2. Mamprusi  3. Gonja  4. Others specify ………………… |  |
| 43 | What is your spouse’s occupation? | 1. Farmer  2. Trader  3. Unemployed  4. Public servant  5. Others specify ……………………. |  |
| 44 | What is your spouse’s religion? | 1. Christianity  2. Muslim  3. Traditionalist  4. Others specify |  |
| 45 | What is your spouse’s monthly income? | GHS…………………..……….. |  |
| 46 | Which area does your spouse live? | 1. Rural  2. Urban  3. Peri urban |  |
| 47 | Does your spouse have children with another woman? | 1. Yes  2. No |  |
| 48 | Are you the only wife of your spouse? | 1. Yes  2. No |  |
| 49 | If no, how many wives does your spouse have? | ……………………………. |  |
| 50 | Do you smoke? | 1. Yes  2. No |  |
| 51 | Does your spouse smoke? | 1. Yes  2. No |  |
| 52 | Do you drink alcohol? | 1. Yes  2. No |  |
| 53 | Does your spouse drink alcohol? | 1. Yes  2. No |  |
| 54 | Have your spouse ever married and divorced | 1. Yes  2. No |  |
| 55 | Does your spouse have multiple sex partners | 1. Yes  2. No |  |
| 56 | Which of the following at times result in misunderstanding? | 1. Money  2. Sex  3. Displaying authoritative  4. Others specify ……………….. |  |
| 57 | Whenever there is disagreement how do you resolve or cope with? | 1. Keep calm  2. Talk back  3. Apologize |  |
| 58 | Have you spouse seen your father beating the wife before? | 1. Yes  2. No |  |
| 59 | Have your spouse seen his father beating the wife before? | 1. Yes  2. No |  |

**Mass media exposure**

| **No.** | **Question** | **Response** |
| --- | --- | --- |
| 60 | How frequent do you listen to radio | 1. Never  2. Always  3. Sometimes |
| 61 | Do you watch television | 1. Yes  2. No |
| 62 | How frequent do you read newspapers or magazine? | 1. Never  2. Always  3. Sometimes |

**Women empowerment**

| **No.** | **Question** | **Response** |
| --- | --- | --- |
| 63 | Who decides on respondent health care | 1. Myself  2. Spouse  3. Both |
| 64 | Who decides on large household purchase? | 1. Myself  2. Spouse  3. Both |
| 65 | Who decides on household purchase for daily needs? | 1. Myself  2. Spouse  3. Both |
| 66 | Who decides on visiting family or relatives? | 1. Myself  2. Spouse  3. Both |
| 67 | Do you have control over your own earnings? | 1.Yes  2. No |
| 68 | Do you have control over your spouse’s earnings? | 1.Yes  2. No |
| 69 | Do you know the amount of money your spouse earns? | 1.Yes  2. No |
| 70 | Who decides on contraceptive usage? | 1. Myself  2. Spouse  3. Both |
| 71 | Who decides on the number of children to have? | 1. Myself  2. Spouse  3. Both |

**Section D: Perspective on IPV**

**Women’s perspective on IPV**

| **No.** | **Question** | **Response** |
| --- | --- | --- |
| 72 | How do you see a woman been abused? | 1. Normal  2. Very terrible |
| 72 | Are you aware that violence against women is against the law | 1. Yes  2. No |
| 73 | Have you/would you have taken any step on abuse | 1. Yes  2. No |
| 74 | What step did you/ would you have taken? | 1. Report to police  2. Report to Pastor  3. Report to Imam  4. Report to family |
| 75 | In case you have ever reported abusive case to authorities what did they do about it? | 1. Nothing  2. They followed up |
| 76 | Was there any change after you have reported? | 1. Yes  2. No |
| 77 | If yes, how was the change? | 1. Positive  2. Negative |
| 78 | If your spouse abuse you, how do you take it? | 1. Very bad  2. Is a sign of love from the man |

**Cultural perspective on IPV**

| **No.** | **Question** | **Response** |
| --- | --- | --- |
| 79 | In your culture is it normal for a man to beat his wife | 1. Yes  2. No |
| 80 | Are your family members going to be happy if you are being abused | 1. Yes  2. No |
| 81 | Will your parents allow you to leave the marriage if you are being abused | 1. Yes  2. No |
| 82 | Will you leave your marriage if you are abused? | 1.Yes  2.No |
| 83 | If NO, Why? | ………………. |
| 84 | How does the society see a woman who left her marriage due to violence? | 1. Normal  2. Mockery |

**Section E: Economic status Assessment Check List**

| **ITEM(s)** | **Please Tick appropriate** |
| --- | --- |
| ***Accommodation ownership*** | 1. Owned [ ]  2. Rented [ ] |
| ***Does any member of your household own agriculture land*** | 1. Yes [ ] 2. No [ ] |
| ***Type of Building*** | 1. Clay house [ ]  2. Single bedroom [ ]  3. Self-contained [ ]  4. More than 3 bedrooms [ ]  5. Storey Building [ ] |
| ***Source of Drinking water for household use*** | 1. Piped  2. Bore well/hand pump  3. Open well  4. Dam  ***Others (Specify)…………………*** |
| ***Type of toilet facility shared by household*** | 1. Flush or pour flush toilet  2. Flush to piped sewer system  3. Flush to septic tank  4. KVIP  5. NO facility/bush/field  Others (specify)……………………. |
| Livestock | 1. Yes [ ] 2. No [ ] |
| Car/truck | 1. Yes [ ] 2. No [ ] |
| Motor bike | 1. Yes [ ] 2. No [ ] |
| Animal drawn carts | 1. Yes [ ] 2. No [ ] |
| Corn mill | 1. Yes [ ] 2. No [ ] |
| A set of furniture | 1. Yes [ ] 2. No [ ] |
| Television | 1. Yes [ ] 2. No [ ] |
| Refrigerator | 1. Yes [ ] 2. No [ ] |
| Washing machine | 1. Yes [ ] 2. No [ ] |
| Computer/Laptop | 1. Yes [ ] 2. No [ ] |
| Sowing machine | 1. Yes [ ] 2. No [ ] |
| DVD | 1. Yes [ ] 2. No [ ] |
| Radio | 1. Yes [ ] 2. No [ ] |
| Mobile phone | 1. Yes [ ] 2. No [ ] |
| Bicycle | 1. Yes [ ] 2. No [ ] |
