# Supplementary material for: Prevalence and Predictors of Intimate Partner Violence During Pregnancy in Northern Ghana: A Cross‐Sectional Study
Source: Health Sci Rep. 2026 Apr 15;9(4):e72351. doi: 10.1002/hsr2.72351 (PMC13083581; doi:10.1002/hsr2.72351)
Supplement: Supplementary file 4 — Supporting File 4 [file HSR2-9-e72351-s004.docx]

**Figure 1: Flow Diagram of Participant Selection and Enrolment**

*Prevalence and Predictors of IPV During Pregnancy in Northern Ghana*

Tamale Metropolis, Ghana | April – May 2023

| **STEP 1: STUDY SETTING** 3 Public Health Facilities — Tamale Metropolis, Northern Ghana (1 Tertiary: Tamale Teaching Hospital + 2 Secondary: Tamale Central & Tamale West Hospitals) Data collection period: April – May 2023 |
| --- |

▼

| **STEP 2: TOTAL ANC ATTENDEES ASSESSED FOR ELIGIBILITY** N = [Total from ANC registers — 490] Source: ANC daily attendance registers at each facility Sampling frame constructed from daily ANC registers; systematic sampling applied (interval = 3) |
| --- |

▼

| **STEP 3: MET ELIGIBILITY CRITERIA** Inclusion criteria met: ✔ Pregnant woman with ANC record book ✔ In a current partnership ≥ 12 months ✔ Age ≥ 18 years ✔ Tamale resident ≥ 6 months ✔ Provided written informed consent | **➤** | **EXCLUDED** **Reasons for exclusion:** ✘ High-risk pregnancy*  (placenta previa, pre-eclampsia,  multiple gestations) ✘ Non-consenting ✘ Absent during data collection:    * Clinical rationale: specialised management,  elevated distress may confound IPV disclosure |
| --- | --- | --- |

▼

| **STEP 4: ENROLLED** **N = 260 pregnant women** Allocation by Probability Proportionate-to-Size (PPS): ● Tamale Teaching Hospital (Tertiary): n = [106] ● Tamale Central Hospital (Secondary): n = [90] ● Tamale West Hospital (Secondary): n = [64]  Note: 260 enrolled > 169 minimum (Cochran formula + 10% non-response rate) Additional enrolment improved statistical power and precision of estimates |
| --- |

▼

| **STEP 5: DATA COLLECTION** Structured questionnaire (adapted from Ghana DHS 2022) Interviewer-administered (Dagbani) or self-administered (English) per participant preference Duration: approximately 20–45 minutes per participant |
| --- |

▼

| **STEP 6: INCLUDED IN ANALYSIS** **N = 260 (all enrolled participants)** Missing data handled by listwise deletion No participants withdrew after enrolment |
| --- |

▼

**PRIMARY OUTCOME: IPV DURING CURRENT PREGNANCY**

| **✔ EXPERIENCED IPV** **n = 156** (60.0%; 95% CI: 54.1%–65.9%) |  | **✘ NO IPV EXPERIENCED** **n = 104** (40.0%; 95% CI: 34.1%–45.9%) |
| --- | --- | --- |

**Note:** *CI = confidence interval; DHS = Demographic and Health Survey; IPV = intimate partner violence; ANC = antenatal care; PPS = probability proportionate to size.*
