# Supplementary figures and images for: Prevalence and Predictors of Intimate Partner Violence During Pregnancy in Northern Ghana: A Cross‐Sectional Study
Source: Health Sci Rep. 2026 Apr 15;9(4):e72351. doi: 10.1002/hsr2.72351 (PMC13083581; doi:10.1002/hsr2.72351)

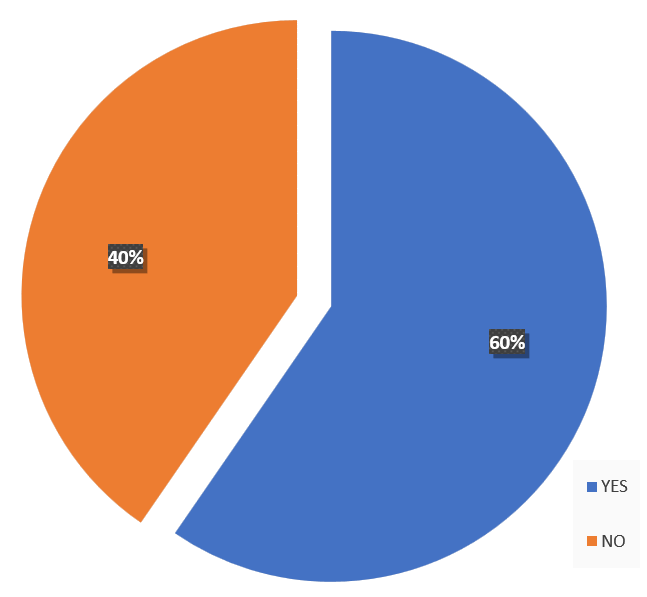


Figure 1: Prevalence of intimate partner violence among women during pregnancy

Supplement: Supplementary file 5 — Supporting File 5 [file HSR2-9-e72351-s001.docx]
